# The Three Glass Test in Cases of Pyuria

**Published:** 1907-12-28

**Authors:** 


### Points in Diagnosis.

### THE THREE-GLASS TEST IN CASES OF PYURIA.

When a patient has pyuria, it is not at all an uncommon thing for a mixed specimen of the urine to be sent to a clinical or bacteriological laboratory for examination and report, without any attempt being made by the patient or his medical adviser to assist the laboratory worker by collecting the urine in successive portions. Sometimes two specimens are sent, one of morning urine, and another of that passed later in the day, but it seldom happens that the urine from the same micturition is sent up in separate portions, with an intimation as to which was passed first and which later. There is a fair reason for this in some cases—the examination will be more costly, the greater the number of portions that have to be investigated separately. This, however, is a very small matter compared to the dangerous errors that may be avoided by what may be called the "two-glass test" or the "three-glass test," as the case may be.

Sir Henry Thompson, as long ago as 1868, tried to direct the attention of the profession to this matter, but his teaching is less followed than it might be. In his own words:—

"Whenever you want a specimen from your patient to examine, do not tell him to send you a bottle of it, passed in the usual way, or you will get a mixture often of doubtful value. What you require is the secretion of the kidneys, plus only anything there may be in the bladder; but you have also to avoid the presence in it of any secretion which originated in the urethra. Make a point of demanding that the patient should first pass two or three tablespoonfuls through the urethra, so as to sweep out whatever may happen to be there (which may be thrown away, or be put into a separate bottle), after which you will get a pure specimen for examination—at any rate, one of which you will know the source. You will have the renal secretion, plus only whatever deposit may be produced in the bladder. Suppose the patient has gleet or chronic prostatitis, there will then be a quantity of muco-purulent matter in the urethra. If all this be carried into one vessel with the urine, how will you determine the different products, and decide, by the eye or by the microscope, what has come from the urethra, what from the prostate, and what from the kidneys? You cannot do it; but if you get rid of the source of error by flushing the urethra, so to speak, that is, by passing the first two or three tablespoonfuls into a wine-glass, while all that follows is passed into a separate vessel, such as a tumbler, you will have in the latter a sample of urine that can be relied upon for examination. If I felt disposed to indulge you with gossip, I could tell you stories of the gravest blunders committed by not attending to this simple point. I can at all events say that I have more than once known a patient treated for pyelitis whose only complaint was a profuse discharge from the urethra. He had sent the urine twice a week to his adviser for examination, in a bottle scrupulously made clean for the purpose, and because a quantity of pus was found in it, was treated during some months for pyelitis, having some symptoms corroborating that view. At length another observer discovered that all the pus came from the urethra, for when the urine was passed in two glasses, the first glass contained all the thick matter, and the remaining urine was clear and healthy; so that, finally, the pyelitis soon disappeared under local treatment of the urethra. Purulent matter originating in the urethra is often mixed with specimens of urine sent for examination, in which case it may be erroneously estimated as albumen by the chemical test; or as pus under the microscope may be supposed to have its origin from the deeper passages."

Sir Henry Thompson therefore advocated the two-glass test. It is important to remember, however, that the last small portion of urine that is expelled usually brings into action the levator ani and compressor urethræ muscles and that these often squeeze out purulent material from the posterior urethra or from the prostate, or both, supposing there were any posterior urethritis or prostatitis in the case. It would be wise, therefore, to collect separately the last few tablespoonfuls of urine as well as the first, and discard them if the specimen to be examined is required to represent the urine from kidneys, ureters and bladder only. In other words, the three-glass test is advocated in cases of pyuria if errors are to be avoided. Some observers go even further than this, but it is sufficient for the present to lay stress upon the main issue only, and to urge that the urine be collected in these cases, not in mixed bulk, but in three portions, which, for the sake of argument, we may style A, B and C.

*Portion A* consists of the first ounce or two of urine, together with any pus which may have been present in the urethra.

*Portion B* consists of the main bulk of the urine, less the first ounce or two and the last ounce or two, and represents the condition of the urine as it is in the bladder.

*Portion C* consists of the last ounce of urine, together with any pus that may have been expressed from the posterior urethra or from the prostate by the squeezing action of the levatores ani and the compressor urethræ muscles.

If portion A contains pus, and portions B and C do not, there is probably a superficial urethritis, but no cystitis and no pyelitis. If portion A contains pus, portion B contains none, and portion C contains pus, there is probably not only a superficial urethritis, but also a deep urethritis in the posterior urethra, and probably prostatitis, but no cystitis or pyelitis. If portions A and B contain no pus, but portion C does, the lesion is probably confined to the posterior urethra and prostate—a chronic posterior urethritis and prostatitis, but no cystitis or pyelitis. If portions A, B and C all contain pus, there is certainly a suppurative lesion in the bladder or in the kidneys, or in both, and there may or may not be urethritis or prostatitis as well.

